# Research progress on the mechanisms of polysaccharides derived from traditional Chinese medicine in the treatment of Parkinson’s disease

**DOI:** 10.3389/fphar.2025.1566479

**Published:** 2025-08-08

**Authors:** Xiangling Zhou, Qi Pan

**Affiliations:** Department of Neurology, People’s Hospital of Dong Xihu, Wuhan, China

**Keywords:** polysaccharides, traditional Chinese medicine, Parkinson’s disease, molecular mechanisms, neuroprotection, research progress

## Abstract

Parkinson’s disease (PD) is currently a highly prevalent neurodegenerative disease. Its pathogenesis is complex, and there is no effective method to prevent the disease progression. Traditional Chinese medicine (TCM) has a unique advantage in treating PD through the approach of syndrome differentiation. TCM prescriptions for PD can reduce Unified Parkinson’s Disease Rating Scale (UPDRS) scores, improve non-motor symptoms, and decrease adverse drug reactions. Bioactive polysaccharides extracted from prescribed Chinese herbs exhibit diverse biological activities due to their wide range of botanical sources. This review summarizes the pharmacological mechanisms of polysaccharides derived from TCM in managing PD, including inhibition of apoptosis, activation of autophagy, regulation of inflammation, anti-oxidative stress, improvement of mitochondrial function, and neuroprotective effects, aiming to provide a theoretical basis for future research and treatment.

## 1 Introduction

Parkinson’s disease (PD) is the second most common neurodegenerative disorder (Tolosa et al., 2021; Steinmetz et al., 2024). First described by British physician James Parkinson in 1817 (Morris, 1989), PD was once considered rare. However, its global prevalence increased by 74% between 1990 and 2016 (Ben-Shlomo et al., 2024). Since the beginning of the 21st century, PD has become more prevalent, driven by aging populations, longer life spans, declining smoking rates, and the by-products of industrialization. The global burden of PD is projected to exceed 12.9 million by 2040, with China expected to have five million cases by 2030, representing nearly half of the global patients (Dorsey and Bloem, 2018; Chinese Society of Parkinson’s Disease and Movement Disorders, 2020). The prevalence of PD is 1.7% in individuals over 65 years old and exceeds 4% in those over 80 years old.

**FIGURE 1:**
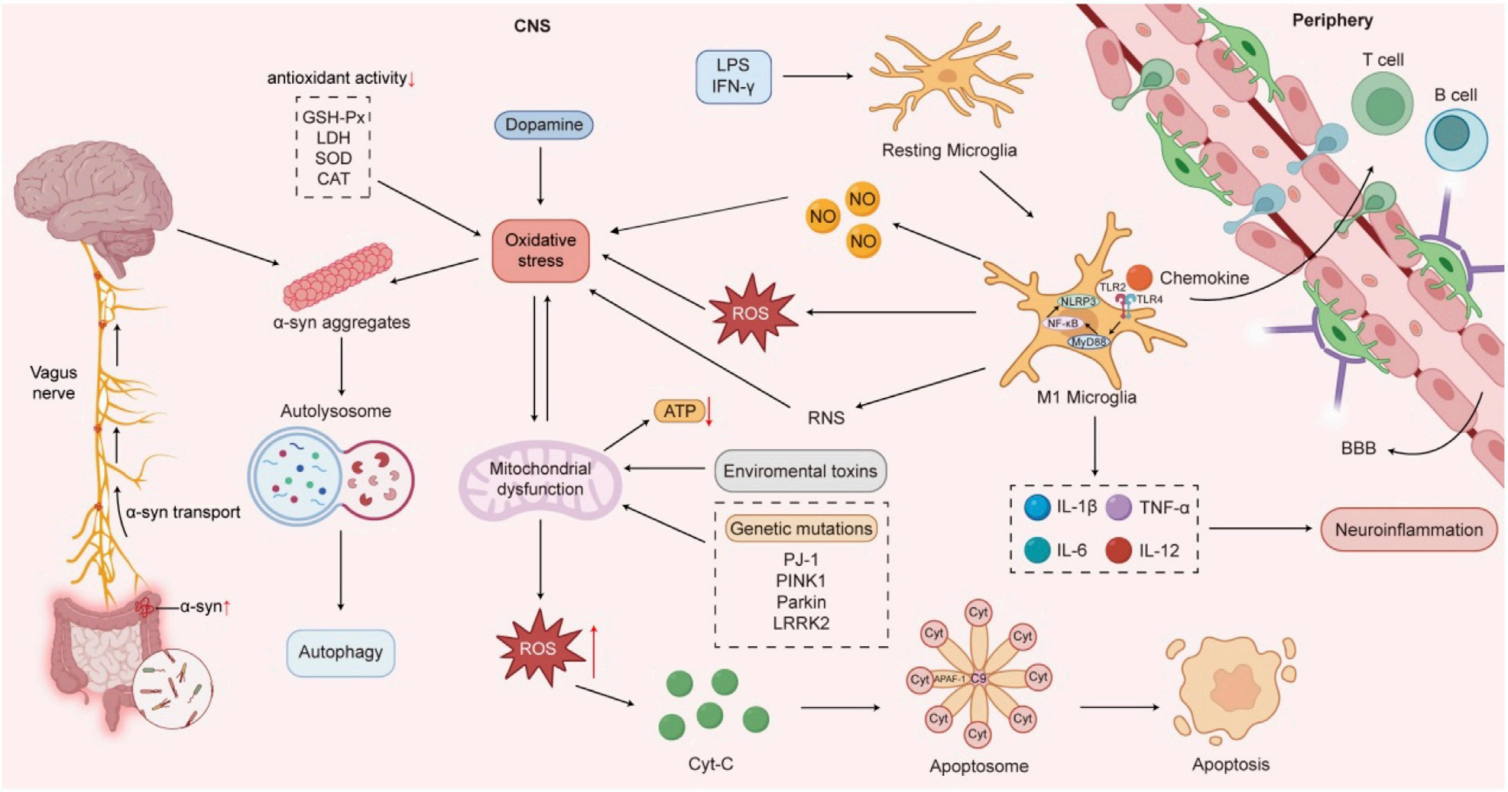
The pathogenesis of Parkinson’s disease.

The pathogenesis of PD involves multiple factors, including age, environmental triggers, hereditary and genetic mutations, neuroinflammation, oxidative stress, mitochondrial dysfunction, abnormal aggregation of α-synuclein (α-syn), lysosomal dysfunction, gut microbiota dysbiosis, and dysregulated α-syn transmission along the gut-brain axis (Figure 1). Current pharmacological treatments face challenges, such as diminishing efficacy and emerging complications as the disease progresses. In contrast, TCM exhibits multi-target mechanisms and enhanced safety profiles, improving both motor and non-motor symptoms while potentially slowing disease progression (Yang et al., 2024).

Polysaccharides derived from TCM demonstrate significant therapeutic potential in PD through diverse biological activities. This paper aims to review the sources and mechanisms of polysaccharides from TCM in PD prevention and treatment to facilitate their further development in this field.

## 2 Sources and components of polysaccharides from traditional Chinese medicine

### 2.1 Sources of polysaccharides

Polysaccharides from Traditional Chinese medicine refer to bioactive substances containing a large number of polysaccharides extracted from traditional Chinese medicine, which have diverse sources from fungi, plants, animals, microorganisms and marine organisms. Polysaccharides from fungi, such as Antrodia camphorata polysaccharides, Chroogomphus rutilus polysaccharides, morchella polysaccharides and bergamot polysaccharides, are typically obtained through fermentation or cultivation in specific media. Plant-derived polysaccharides are the most extensively studied, including *Lycium barbarum polysaccharides* (LBP), g*inseng polysaccharides*, *Angelica sinensis polysaccharides* (ASP), *Astragalus polysaccharides* (APS), *Poria cocos polysaccharides*, and *Dendrobium polysaccharides* (DP) etc. They are usually extracted from Chinese medicinal herbs through traditional methods like water extraction and alcohol precipitation. Animal polysaccharides, such as heparin, chitin and chondroitin sulfate, are usually obtained by extracting from animal organs and tissues. Microbial polysaccharides, such as hyaluronic acid and gel polysaccharides, are primarily produced through microbial fermentation. Marine biogenic polysaccharides, such as spirulina and wakame, are derived from marine organisms through extraction.

### 2.2 Components of polysaccharides from traditional Chinese medicine

The structure of polysaccharides from TCM is complex, and the extraction and purification process have not been fully standardized. Polysaccharides from TCM are mainly composed of 3–10 monosaccharides, including glucose, arabinose, rhamnose, xylose, fucose, galacturonic acid and glucuronic acid, with neutral polysaccharides being the main type. The specific components of polysaccharides from TCM are shown in Table 1.

**TABLE 1:** Monosaccharide composition of polysaccharides from TCM for prevention and treatment of PD.

Polysaccharides Name	Monosaccharide composition	Number of monosaccharides	Type of monosaccharides	Ref
Angelica sinensis polysaccharides	glucose, arabinose, rhamnose, glucuronic acid, galacturonic acid	5	neutral sugar, acidic sugar	Zhao et al. (2012)
Bergamot polysaccharides	rhamnose, xylose, mannose, glucose, galactose	5	neutral sugar	Peng et al. (2018)
Rehmanniae glutinosa polysaccharides	glucose, rhamnose, arabinose, galactose, galacturonic acid	5	neutral sugar, acidic sugar	Zhou Y. et al. (2021)
Lycium barbarum polysaccharides	glucose, arabinose, galactose, mannose, xylose, rhamnose, fucose, galacturonic acid, glucuronic acid	9	neutral sugar, acidic sugar	Tian et al. (2019)
Polygonatum sibiricum polysaccharides	galactose, arabinose, rhamnose, xylose, glucose	5	neutral sugar	Wang et al. (2019)
Astragalus membranaceus polysaccharides	rhamnoose, arabinose, xylose, starch, galactose, glucuronic acid, galactose	7	neutral sugar, acidic sugar	Zheng et al. (2020)
Cistanche polysaccharides	mannose, rhamnose, galacturonic acid, glucose, galactose, arabinose, fructose	7	neutral sugar, acidic sugar	Cheng et al. (2023)
Dendrobium polysaccharides	mannose, glucose, rhamnose, arabinose, ribose, galactose, xylose, glucuronic acid, galacturonic acid, fructose	10	neutral sugar, acidic sugar	Yue et al. (2020)
Chroogomphus rutilus polysaccharides	mannose, glucose and galactose	3	neutral sugar	Zhao et al. (2021)
Schisandra chinensis polysaccharides	mannose, galacturonic acid, glucose, galactose, arabinose	5	neutral sugar, acidic sugar	Zhang et al. (2022)
matsutake polysaccharides	mannose, glucose, galactose, xylose, fucose	5	neutral sugar	Wang et al. (2023)
Asparagus polysaccharides	rhamnose, arabinose, galactose, glucose, xylose, mannose, galacturonic acid	7	neutral sugar, acidic sugar	Mu et al. (2022)
Poria cocos polysaccharides	glucose, fucose, arabinose, xylose, mannose, galactose	6	neutral sugar	Xu et al. (2022)
Antrodia camphorata polysaccharides	galactose, glucose, mannose	3	neutral sugar	Wang (2024)

## 3 Biological activities of polysaccharides from common traditional Chinese medicine

The composition of polysaccharides is relatively complex. For the same traditional Chinese medicine, due to different varieties, origins and growth time, the extracted polysaccharide components have slight differences in molecular weights, monosaccharide composition, glycosidic bonds and biological functions (Zeng et al., 2019). The structural complexity of polysaccharides leads to the diversity of their biological activities. Because of their unique sugar chain structures and functional groups, different polysaccharides can interact with biological macromolecules, such as receptors and enzymes, to exert their therapeutic effects. Polysaccharides have various biological activities, such as antiviral (Hao et al., 2024), anti-inflammatory, anti-oxidative stress (Huang et al., 2017), anti-tumor (Wang et al., 2024) and immunomodulatory effects (Li W. et al., 2020). A recent study has found that polysaccharides from traditional Chinese medicine also exhibit significant anti-aging effects in animal models (Song et al., 2024).

Statistical analysis on the frequency of use of individual herbs shows that cooked *Rehmannia glutinosa* (Gaertn.) Libosch. ex DC. [Orobanchaceae](processed), *Astragalus mongholicus Bunge* [Fabaceae], Gastrodia elata Blume [Orchidaceae], *A. sinensis* (Oliv.) Diels [Apiaceae], *L. barbarum* L.[Solanaceae], *P. cocos* (Schw.) Wolf. [Polyporaceae], *Cistanche deserticola Ma* [Orobanchaceae], and *Salvia miltiorrhiza Bunge* [Lamiaceae], etc. are used more frequently. Polysaccharide components of many Chinese medicines are important active agents for the treatment of PD models, such as those possessing the activity of mitigating oxidative stress and modulating inflammatory pathways.

### 3.1 Astragalus membranaceus polysaccharides

*Astragalus mongholicus Bunge* [Fabaceae] is the root of the leguminous plant Astragalus membranaceus (Fisch.) BGe.var.mongholicus (Bge.) Hsiao or Astragalus membranaceus (Fisch.) Bge. APS can be used as an immune promoter or regulator, and has pharmacological effects, such as anti-inflammatory, immune modulatory, and neurodegeneration-delaying (Du et al., 2022). The chemical characterization of APS (e.g., branching degree, molecular weight) is closely related to its immunomodulatory functions, while its bioavailability and bioactivity can be enhanced through structural modifications (e.g., sulfation) or processing methods (e.g., honey-processing). Specifically, APS with highly branched chains, low-to-medium molecular weights, large surface areas, or selenated modifications significantly promotes immune cells’ activities. Additionally, honey-processed APS with low-to-medium molecular weights exerts anti-inflammatory activities by regulating immune cells, immune organs and immune factors (Zhao et al., 2024).

### 3.2 Angelica sinensis polysaccharides

Angelica sinensis is derived from the dried root of the umbelliferous plant Angelica sinensis (Oliv.) Diels. It has a wide range of pharmacological effects, including hematopoietic promotion, immune enhancement, antitumor activity, and antioxidant properties (Jin et al., 2012). Studies have linked these biological activities to the structural features of its polysaccharides, showing that a high content of galacturonic acid has stronger protection and anti-oxidation effect on macrophages (Jin et al., 2022).

### 3.3 Lycium barbarum polysaccharides

Goji berries are tonic medicines used for deficiency syndromes. As the dried mature fruits of the Solanaceae family, LBP are water-soluble compounds with multiple biological activities, including immune enhancement, anti-aging, anti-tumor effects, free radical scavenging, anti-fatigue, anti-radiation, and hepatoprotective and reproductive system protection. Structural analysis reveals that LBP1 (Mw = 1.207 × 10^6^) exhibits significantly stronger immunomodulatory activity than LBP2 (Mw = 1.25 × 10^5^) (Wang Y. et al., 2021). Crude LBPs containing sulfated groups, carboxyl moieties, and higher protein content demonstrate superior antioxidant and immunoregulatory properties compared to purified LBPs (Luo et al., 2004; Wang et al., 2010).

### 3.4 Rehmanniae glutinosa polysaccharides

This herb is the dried root or freshly processed product of *Rehmannia glutinosa* (Scrophulariaceae family). Polysaccharides from the cooked *Rehmannia* are one of the major active components, exhibiting pharmacological effects, such as antioxidant, anti - aging, anticancer and immunomodulatory activities (Bian et al., 2023). Polysaccharides of the cooked rehmannia have complex compositions, and their content after processing and steaming is higher than that in the raw *Rehmanna* glutinosa (Geng, 2023). *Rehmannia Radix* polysaccharides (RRPs) with high content of total lactose and glucose show strong biological activities. For example, the extract RGP70 with high galactose content exhibits the highest antioxidant and anti-aging activity (Huang et al., 2024). RRPs containing high levels of glucuronic acid also demonstrate antioxidant activities (Ren et al., 2022).

### 3.5 Poria cocos polysaccharides

This herb is the dried sclerotium of the Tuckahoe fungus. *Poria cocos Pachyman* (PCP) is the main active component, accounting for about 70%–90% of the sclerotia’s dry weight. It has biological functions, such as anti-oxidation, anti-aging, anti-inflammation, immune regulation, antibacterial activity, hepatoprotection and anti-cancer effects (Li et al., 2022). The phagocytotic capacity of RAW264.7 cells is enhanced to the greatest extent when PCP is combined with lentinan (Dong et al., 2021).

### 3.6 Cistanche polysaccharides

This herb belongs to a tall herbaceous plant genus of Cistanche Hoffing. et Link and the family of Orobanchaceae. It has multiple pharmacological effects including improving the learning and memory ability, regulating gut microbiome, anti-inflammation, anti-aging, anti-oxidation (Zhang et al., 2016), anti-depression, regulating immune functions, moistening the bowel, managing osteoporosis, and protecting the body from chronic alcoholic liver disease (Guo et al., 2016), ischemic stroke and oxidative stress injury (Xue et al., 2024). Polysaccharides of different molecular weights promote cell proliferation at low concentrations and inhibit cell proliferation at high concentrations (Gao, 2022).

## 4 Clinical application and mechanisms of Chinese medicine prescriptions

At present, many formulations containing polysaccharides from Chinese herbs have been clinically proven to improve the motor and non-motor symptoms of PD patients. Mechanistic studies further confirmed that these formulations play a role through anti-oxidation, anti-apoptosis, anti-inflammatory, inhibition of α-syn aggregation and other pathways. The mechanisms of these formulations directly correlate with the pharmacological properties of polysaccharides. Three clinically validated prescriptions, such as Cong Rong Shu Jing Granules and Chaihu Jia Longgu Muli Decoction, demonstrate these synergistic effects.

### 4.1 Cong Rong Shu Jing compound (CRSJ)

Chen et al. (2019), Chen et al. (2024) evaluated the efficacy and safety of Cong Rong Shu Jing granules in substantia nigra metabolites in PD patients. Compared with the control group, the treatment group demonstrated significant reductions in MDS-UPDRS I, MDS-UPDRS III, PDQ-39, and NMSS scores, indicating that CRSJ can improve both motor and non-motor symptoms as well as PD patients’ quality of life. Magnetic resonance spectroscopy (MRS) analysis showed that the N-acetylaspartic acid/creatine (NAA/Cr) ratio in the mild side of substantia nigra in the treatment group was increased. Since NAA reflects neuronal activity in the brain and Cr serves as an internal benchmark to evaluate metabolic rates, these findings suggest that CRSJ granules may improve the symptoms of PD patients by increasing the activity of substantia nigra neurons. Safety assessments, including blood tests, urinalysis, liver/kidney function tests, electrocardiograms and recorded adverse events, showed that all patients had good safety indicators. The dosage of Madopar decreased in the CRSJ group. A study (Fang and Cai, 2021) has compared the effect of CRSJ granules on improving sleep with Western medicine. The results showed significantly lower scores on both the Hamilton Depression Scale (HAMD) and Pittsburgh Sleep Quality Index (PSQI) in the experimental group compared to the control group.

A PD mouse model was established with MPTP (1-methyl-4-phenyl-1, 2, 3, 6-tetrahydropyridine). Their results showed as follows (Cai, 2013): the contents of DA and its metabolites (HVA and DOPAC) in the CRSJ group were significantly higher than those in the model group, the number of tyrosine hydroxylase (TH) positive cells in the CRSJ group was significantly increased. Under electron microscopy, most neurons still maintained their normal morphology. Compared with the model group, Caspase-3, Fas/FAS-L and Bax levels in the CRSJ group were significantly reduced, while Bcl-2 was increased. Bcl-2 is a protective factor against apoptosis, indicating that nerve cells in the CRSJ group initiated self-protection. This formula could significantly reduce the apoptosis of nigrostriatal neurons in PD animal models, alleviate structural damage to nigrostriatal neurons, reverse reduced dopamine levels and increase the level of the TH in dopamine anabolism.

CRSJ granule can increase the level of cerebral dopamine neurotrophic factor (CDNF) and the mesencephalic astrocyte-derived neurotrophic factor (MANF). It also activates the phosphoinositide 3-kinase (PI3K)/protein kinase B (AKT) pathway-related proteins to inhibit cell apoptosis. CRSJ granule can also decrease the expression of α-syn and the level of endoplasmic reticulum stress (ERS) related proteins, such as glucose regulatory protein 78 (GRP78), inositol-requiring enzyme one (p-IRE1α), apoptosis signal-regulating kinase 1 (ASK1), c-Jun N-terminal kinase (p-JNK) and caspase-12. This leads to reduced ERS in PD rats, inhibited apoptosis of dopaminergic (DA) neurons, increased numbers of TH positive cells, and improved the motor functions of mice (Lin et al., 2020; Xu et al., 2020).

### 4.2 Chaihu Jia Longgu Muli Decoction

Tu and Cai, (2017) conducted a randomized controlled study and found that Chaihu Jia Longgu Muli Decoction improved the depressive symptoms in PD patients with liver-qi depression (PDD). The decoction showed early-stage clinical efficacy by improving daily living ability and reducing TCM syndrome scores in PDD patients. The mechanism may be related to the increased serum levels of 5-HT and NA. Xiao et al. (2021) applied Chaihu Jia Longgu Muli Decoction to treat sleep disorders in PD patients. Their study revealed a significant increase in Parkinson’s Sleep Scale score (PDSS) among 30 patients after treatment compared to baseline.

Another study (Yu et al., 2021) found that Chaihu Jia Longgu Muli Decoction reduced Ashworth scores (indicating improved muscle tone) and elevated scores of life quality, MoCA and MMSE in PD patients with dementia, compared to a western medicine-only group. After treatment, the decoction group exhibited lower levels of S100β, Aβ, and neuron-specific enolase (NSE) biomarkers. These results suggest that Chaihu Jia Longgu Muli Decoction may reverse cognitive decline and improve clinical outcomes in dementia-associated PD.

A study (Hu et al., 2020) reported that Chaihu Jia Longgu Muli Decoction combined with acupuncture treatment increased serum superoxide dismutase (SOD) levels, decreased malondialdehyde (MDA) and glial fibrillary acidic protein (GFAP) levels, and ameliorated patients’ cognitive and motor functions as shown by UPDRS. These effects may result from suppressing oxidative stress-induced damage to DA neurons.

Another study (Liu et al., 2022) demonstrated that Chaihu Jia Longgu Muli Decoction could improve the depression state of PD rats, evidenced by increased total horizontal movement distance, prolonged central area exploration time in the open field test, and enhanced pole-climbing performance. The number of DAergic neurons in the substantia nigra, enlarged neuronal Soma size, and increased striatal DA and serotonin (5-HT) levels. The treatment significantly downregulated α-Syn expression, upregulated LC3-II/LC3-I and p-AMPK/AMPK ratios, while inhibiting p-mTOR/mTOR signaling. The therapeutic mechanism likely involves activating the AMPK/mTOR pathway to enhance autophagy-mediated clearance of pathological α-Syn aggregates.

### 4.3 Shudi Pingchan decoction

A Multi-clinical study by Yuan et al. (2010) showed that Shudi Pingchan decoction could significantly alleviate motor symptoms of PD patients manifested by decreased UPDRS-II and UPDRS-III scores, prolonged on-period and shortened off-period with no apparent side effects observed. This indicates that the decoction potentiates therapeutic efficacy while attenuating drug-induced toxicity in PD management.

Another study on this herbal decoction and repetitive transcranial magnetic stimulation (rTMS) (Ye et al., 2017b) demonstrated a good therapeutic effect on PD patients. The high-frequency rTMS group showed superior UPDRS-III score reductions *versus* controls at 8-week and 12-week. Non-motor improvements included lower HAMA, Hamilton Depression Rating Scale (HAMD), SCOPA-AUT and Parkinson’s Disease Questionnaire-39 (PDQ-39) scores, alongside higher PDSS (all p < 0.05). Both trials confirmed the decoction’s safety profile, with adverse event incidence comparable to controls and only mild transient reactions reported.

In an animal study, Ye et al. (2016) observed that Shudi Pingchan granule significantly reduced rotational behavior and the duration of spins in PD rats, and reduced substantia nigra apoptosis. Mechanistically, the decoction inhibited ERK1/2 phosphorylation at Thr202/Tyr204, attenuated nuclear translocation of ERK1/2, and subsequently decreased the Bax/Bcl-2 ratio while upregulating TH expression. These changes are related to neuronal survival and apoptosis. Shudi Pingchan granules can also inhibit α-syn overexpression (Ye et al., 2017a), attenuate the injury to dopaminergic neuron, regulate the imbalance between the direct and indirect pathways mediated by D1 and D2 receptors and effectively alleviate PD symptoms. A summary of mechanistic and clinical studies on TCM formulae can be found in Table 2.

**TABLE 2:** Mechanistic and clinical studies on TCM formulae.

Name	Composition	Mechanisms	Ref	Clinical effect	Ref
Congrong Shujing decoction	Cistanche deserticola Ma [Orobanchaceae]; Polygonatum sibiricum Redouté [Asparagaceae]; Salvia miltiorrhiza Bunge [Lamiaceae]; Paeonia lactiflora Pall. [Paeoniaceae]; Paeonia × suffruticosa Andrews [Paeoniaceae]	increase DA, HVA and DOPAC, increased number of TH-positive cells, decrease Caspase-3, Fas/Fas-L and Bax, increase Bal-2 and activate the PI3K/AKT signaling pathway	Cai, (2013)	decrease MDS-UPDRS I, MDS-UPDRS III, PDQ-39, and NMSS scores, increase NAA/Cr ratio in less inflicted SN on MRS, decrease dosage of Madopar	Chen et al. (2019); Chen et al. (2024)
		decrease GRP78, p-IRE1α, ASK1, p-JNK, and caspase-12	Lin et al. (2020) Xu et al. (2020)	decrease HAMD and PSQI scores	Fang and Cai (2021)
Chaihu plus Longggu Muli decoction	Bupleurum chinense DC. [Apiaceae]; Scutellaria baicalensi Georgi [Lamiaceae]; Codonopsis pilosula (Franch.) Nannf.[Campanulaceae]; Neolitsea cassia (L.) Kosterm.[Lauraceae]; Wolfiporia cocos (F.A. Wolf) Ryvarden & Gilb; Pinelliae Rhizoma Praeparatum Cum Alumine [Araceae]; Rheum palmatum L. [Polygonaceae](processed); Mastodi Ossis Fossilia; Ostrea gigas Thunberg; Haematitum; Ziziphus jujuba Mill.[Rhamnaceae]; Zingiber officinale Roscoe [Zingiberaceae]	activate the AMPK/mTOR signaling pathway to promote degradation of abnormally aggregated α-Syn	Liu et al. (2022)	improve the ADL of PDD patients, decrease TCM syndrome score	Tu and Cai, (2017)
		decrease S100β, β-amyloid and NSE	Yu et al. (2021)	increase PDSS score	Xiao et al. (2021)
		upregulate cAMP/PKA/CREB/BDNF signaling pathways, restore expression of BDNF and its receptor tyrosine kinase B	Zhao et al. (2023)	increase MMSE and MoCA scores	Yu et al. (2021)
				increase serum SOD, and decrease MDA and GFAP	Hu et al. (2020)
				Decrease HAMA score, increase serum 5-HT and MHPG	Jia and Li, (2022)
Shudi Pingchan granule	Rehmannia glutinosa (Gaertn.) Libosch. ex DC. [Orobanchaceae]; Lycium barbarum L.[Solanaceae]; Taxillus chinensis (DC.) Danser [Loranthaceae]; Gastrodia elata Blume [Orchidaceae]; Paeonia lactiflora Pall. [Paeoniaceae]; Bombyx Batryticatus; Complete Scorpio; Scolopendra subspinipes mutilans L.Koch[Scolopendridae]; Arisaema amurense Maxim. [Araceae]; Acorus gramineus Aiton [Acoraceae]; Polygala tenuifolia Willd. [Polygalaceae]; Salvia miltiorrhiza Bunge [Lamiaceae]	decrease apoptosis in SN, inhibit activation of the ERK signaling pathway	Ye et al. (2016)	decrease MDS-UPDRS score, HAMA, HAMD, PDQ-39, SCOPA-AUTscores,improve PDSS score	Yuan et al. (2010); Ye et al. (2017b)
		inhibit overexpression of α-syn	Ye et al. (2017a)		

MDS-UPDRS, Movement Disorder Society- Unified Parkinson Disease Rating Scale; NMSS, non-Motor Symptom Rating Scale; PSQI, Pittsburgh Sleep Quality Scale; SCOPA-AUT, autonomic symptoms scale; PDSS, Parkinson’s disease Sleep Scale; HAMA, Hamilton Anxiety Scale; HAMD, Hamilton Depression Scale; MMSE, Mini Mental State Scale; MoCA, Montreal cognitive assessment; PDQ-39, the quality of the survival questionnaire to evaluate Parkinson’s disease; ADL, Activity of Daily Living.

## 5 Anti-PD mechanisms of traditional Chinese medicine polysaccharides

### 5.1 Inhibition of apoptosis

Dopaminergic neurons are a type of highly differentiated and non-renewable cell type. Due to their high energy demands, they are particularly vulnerable to cell death signals and more susceptible to degeneration. Currently, there are over ten known cell death pathways, among which the apoptosis of dopaminergic neurons is considered an important mechanism underlying PD. A notable feature of apoptosis is the formation of apoptotic bodies. Apoptosis primarily occurs via three pathways (Yang X. et al., 2018): the death receptor pathway, the mitochondrial pathway, and the endoplasmic reticulum pathway. The mitochondrial pathway, also known as the intrinsic apoptotic pathway, operates as follows: under the pro-apoptotic stimulation, the permeability of the outer mitochondrial membrane changes, and the mitochondrial permeability transition pore opens, leading to the release of cytochrome C (Cyt C) from the mitochondrial intermembrane space into the cytosol. The released Cyt C combines with apoptotic protease activating factor-1 (Apaf-1) and procaspase-9 to form an apoptotic complex, thereby activating caspase-9 and caspase-3, ultimately triggering apoptosis (Wu and Bratton, 2013). The BCL-2 protein family plays a crucial role in regulating mitochondrial membrane permeability and is a core factor in modulating the mitochondrial apoptotic pathway. Key members include pro-apoptotic proteins, such as BAX and anti-apoptotic proteins, such as BCL-2. When apoptotic signals are activated, BAX undergoes mitochondrial translocation, inserts into the mitochondrial membrane, and polymerizes to form transmembrane channels. This process increases mitochondrial membrane permeability, facilitating the release of Cyt C. BCL-2 inhibits the homodimerization of BAX and its impact on mitochondrial membrane permeability by forming heterodimers with BAX.

The apoptotic pathway is precisely regulated by multiple signaling pathways (He et al., 2022), including the p38 MAPK, JNK/SAPK, Notch, PI3K/AKT/mTOR, and Nrf2/ARE pathways. These pathways form an intricate regulatory network that collectively controls the initiation and progression of apoptosis.

Polysaccharides, such as LBP and ASP, enhance anti-apoptotic mechanisms through reducing reactive oxygen species (ROS) accumulation, inhibiting Cyt-C release, thereby suppressing activation of the intrinsic apoptotic pathway. The neurotoxin MPP^+^ inhibits mitochondrial complex I, elevating ROS accumulation and triggering Cyt-C release. This cascade triggers Cyt-C release from mitochondria, which sequentially activates Caspase-9 and Caspase-3, while dysregulating Bcl-2 family proteins: pro-apoptotic Bax increases, whereas anti-apoptotic Bcl-2 decreases. LBP scavenges ROS and stabilizes mitochondrial integrity, thereby blocking Cyt-C translocation to the cytoplasm and subsequent caspase activation (Wang and Han, 2021). After pretreating SH-SY5Y cells with LBP for 1 h, it was observed that LBP downregulated MPP^+^-triggered overexpression of pro-apoptotic proteins, ultimately, significantly reversed MPP^+^-induced apoptosis. This study utilized SH-SY5Y cells but lacked validation using primary neurons or co-culture with glial cells. LBP pretreatment lasted only 1 h and whether longer pretreatment durations would enhance the protective effect was not verified. Similarly, ASP treatment (Zhang et al., 2024) attenuated astrocytic damage and apoptosis in MPTP-injected mice by modulating Bax/Bcl-2/caspase-3, thereby mitigating MPTP-induced PD-like behavioral and molecular alterations. Consistent with molecular changes, behavioral tests demonstrated that ASP significantly improved limb grip strength and latency time in MPTP-treated mice, alleviating motor deficits in PD models. This experiment evaluated motor function only using a grip strength meter and rotarod apparatus, core behavioral phenotypes for PD, such as gait analysis or open field test, were not included.

**FIGURE 2:**
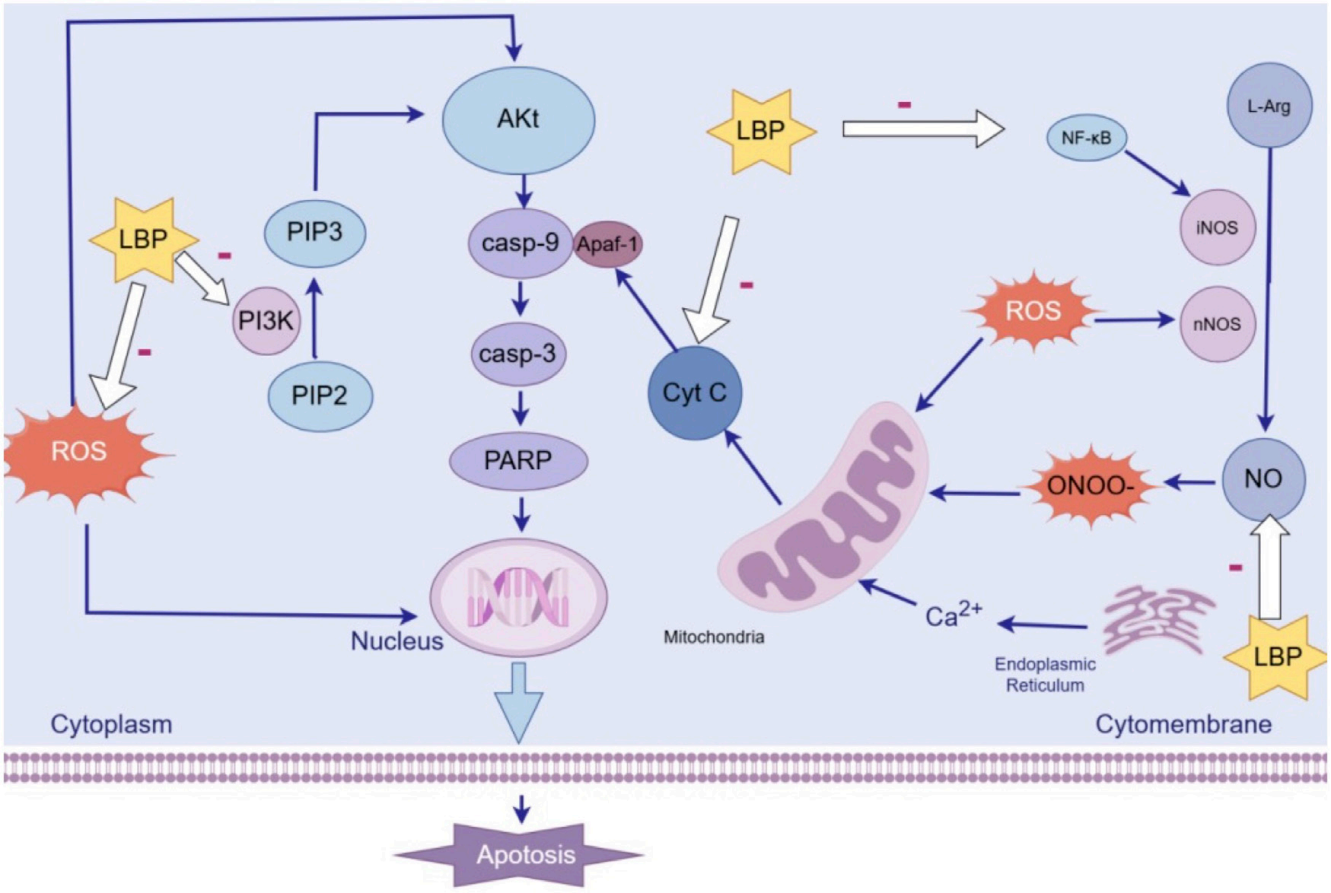
Mechanisms underlying the anti-apoptosis effect of LBP.

The PI3K/Akt pathway is an important pathway of anti-apoptotic signal transduction mechanism, which can protect cells from being stimulated by many apoptotic signals. When PI3K is activated, it phosphorylates PIP2 to PIP3 and promotes the phosphorylation of Akt. Phosphorylated Akt can inhibit the expression and activation of various pro-apoptotic proteins, thereby exerting anti-apoptotic effects. It was shown that LBP (Li P. et al., 2020) could reduce the apoptosis rate of PC12 cells induced by MPP+. After pretreatment with a PI3K inhibitor LY 294002, the protective effect of LBP was blocked. These findings indicate that LBP activates the PI3K/Akt pathway to reduce apoptosis and protect neurons. Mechanisms underlying the apoptosis effect of LBP is presented in Figure 2.

Another study showed (Li et al., 2016) that ASP alleviated the degeneration of dopaminergic neurons and defects in food sensing behavior in nematodes treated with 6-OHDA, and also prolonged the lifespan of nematodes exposed to 6-OHDA. Real time quantitative PCR analysis showed that among programmed cell death related proteins like CED-4, CED-3, CED-9, and EGL-1, ASP significantly reduced the mRNA level of EGL-1 in *Caenorhabditis elegans*, suggesting the role of ASP in regulating the expression of programmed cell death genes. Research has shown that (Wang and Han, 2021) hypoxia induces an increase in the expression of urothelial cancer associated antigen 1 (UCA1) in neurons. UCA1 can promote the progression of PD by regulating the SNCA gene, and its small interfering RNA (si-UCA1) is used for gene silencing. LBP + si-UCA1 is more effective in improving cell survival and reducing apoptosis rate than LBP or si-UCA1 alone, indicating a synergistic anti apoptotic effect of LBP and si-UCA1.

### 5.2 Anti-oxidative stress

During normal life activities, free radicals are constantly produced in the body. Cellular antioxidant defense systems efficiently neutralize these radicals, thereby protecting cells from oxidative damage. However, when the dynamic balance between the oxidation and antioxidation systems is disrupted-particularly when excessive reactive oxygen species (ROS) production overwhelms the antioxidant clearance capacity, oxidative stress occurs. Excessive ROS induces macromolecular structural damage, ultimately leading to cell dysfunction. Notably, dopaminergic neurons in the substantia nigra are highly susceptible to ROS-mediated oxidative injury, a key pathological mechanism in PD.

Studies have shown that the oxidative stress factors leading to PD mainly include the following aspects (Zhu et al., 2019): Firstly, mitochondrial dysfunction, impaired mitochondrial complex I activity substantially increases ROS generation; Secondly, neuroimmune inflammatory responses induce the massive release of pro-inflammatory cytokines (e.g., TNF-α, IL-1β, IL-2), enhances iNOS activities, promoting ROS and ONOO- formation (Przedborski and Dawson, 2001). In addition, abnormal dopamine metabolism, iron accumulation, calcium ion overload, and compromised antioxidant defense system - manifested as reduced activity of antioxidant key enzymes, such as glutathione peroxidase (GSH-Px), superoxide dismutase (SOD), catalase (CAT), lactate dehydrogenase (LDH), and vitamin C (VC).

From a genetic perspective, mutations in mitochondrial quality control genes (e.g., DJ-1, PINK1, and Parkin) disrupt dopaminergic neuron mitochondrial homeostasis (Hu et al., 2021), initiating oxidative cascades. Clinical studies also show that levels of inflammatory factors (IL-6) and oxidative stress markers (SOD, MDA, NOS) in patients with PD are significantly higher than healthy controls (Nagatsu et al., 2000). These results further confirm the role of oxidative stress in the pathogenesis of PD.

Treatment of PC12 cells (a cell line derived from rat pheochromocytoma) with 6-OHDA significantly reduced cell survival and induced typical apoptotic features, such as pyknosis, karyorrhexis, and formation of apoptotic bodies. Excessive ROS accumulation occurs intracellularly. ROS, as the initial trigger of apoptosis, activates p38 mitogen-activated protein kinase (MAPK), induce scaspase-9 and caspase-3 activation, inhibits Akt phosphorylation, and ultimately induces apoptosis. LBP effectively suppressed intracellular ROS generation, blocked p38 activation, reduced 6-OHDA-induced cleavage of caspase-3, and thereby attenuated apoptotic cell death (Gao et al., 2014).

Polysaccharides from TCM significantly inhibit nitric oxide (NO) accumulation and NF-κB activation. The peroxynitrite (ONOO^−^) generated from NO metabolism can Inhibit mitochondrial complexes, induce Ca^2+^ release from the endoplasmic reticulum, trigger Cyt C release, activate apoptosis. Neuronal nitric oxide synthase (nNOS) and inducible nitric oxide synthase (iNOS) are the catalytic enzymes for NO production. 6-OHDA causes overexpression of nNOS and iNOS in PC12 cells, increases intracellular NO and its metabolites, raises Ca^2+^ levels, activates NF-κB in apoptosis-associated signaling pathways, and upregulates p53. After pretreating PC12 cells with different concentrations of LBP, intracellular Ca^2+^ levels decreased, and the expression of nNOS and iNOS as well as the activation of NF-κB were suppressed in a dose-dependent manner (Gao et al., 2014). This indicates that LBP prevents 6-OHDA-induced cell apoptosis by blocking nNOS and iNOS expression, and downregulating the intracellular Ca^2+^and NF-κB pathways. This study used ELISA to measure protein-bound compound to assess the level of nitrosative stress mediated by nitrogen oxides, which is susceptible to non-specific interference. If validated with immunohistochemistry or Western blotting, the conclusion would have greater specificity.

Chen et al. (2018) used LBP to study the impact on antioxidant capacity of brain tissue in Parkinson’s model mice. Their findings revealed a paradoxical dissociation between behavioral outcomes and neuroprotective efficacy: LBP-treated (50 mg/kg, 100 mg/kg) mice exhibited a significant improvement in pole-climbing scores compared to the MPTP group, the pole - climbing scores of LBP - treated mice remained lower than those of the L - DOPA group. This suggests that while LBP partially alleviates motor deficits, its symptomatic efficacy is statistically inferior to conventional L-DOPA therapy. However, the number of tyrosine hydroxylase-positive (TH+) neurons in the substantia nigra pars compacta (SNpc) of the LBP group was larger than that of the MPTP model and the L-DOPA group, suggesting direct neuroprotection. The activities of antioxidant enzymes SOD, GSH Px, and CAT in the LBP group were significantly increased compared to the MPTP group or the L-DOPA group, while the content of oxidative product MDA, a lipid peroxidation marker, was significantly decreased compared to the above two groups. These results highlight LBP’s unique disease-modifying potential through antioxidant and neuroprotective synergy, contrasting with L-DOPA’s transient symptomatic relief. However, the incomplete behavioral recovery implies that dose optimization or combinatorial therapies may be required for clinical translation. The article proposes that LBP protects neurons through antioxidant effects, but it does not specify the pathways and lacks evidence at the molecular level.

When APS was used in combination with L-dopa, the content of glutathione (GSH), activities of the GSH-Px and SOD in L-dopa-induced astrocytes were similar to those of the blank control group, and the content of MDA was not significantly higher than that in the blank control group, and both were better than those in the L-dopa alone group. These reactions were evident 4 h after administering these compounds (Li et al., 2006), indicating that the protective effect of APS was time-dependent. Mechanism studies have shown that L-DOPA produces a large number of free radicals during metabolism, leading to oxidative stress and toxic effects on various neurons. ASP can effectively eliminate free radicals and protect astrocytes treated with L-DOPA from injury by increasing the activity of antioxidant enzymes. Similarly, this study has not explored the specific signaling pathway through which APS modulates oxidative stress levels, which would require further investigation. It is recommended that subsequent research designs quantitatively analyze the key molecular targets of APS through Western blotting and ROS fluorescent probes.

When Gao et al. (2015b) used sulfated Poria polysaccharides (SP) to treat MPP + pretreated cells, it was observed that polysaccharides significantly increased the activity of intracellular antioxidant enzymes (such as GSH Px, SOD, and CAT), while reducing the content of oxidative products (such as hydrogen peroxide-H_2_O_2_ and MDA), thereby reducing the degree of cellular oxidative injury. Further *in vivo* experiments showed that injecting MPTP with SP (Gao et al., 2015a) or Ganoderma lucidum polysaccharides (Yang et al., 2017) significantly increased the activity of antioxidant enzymes and the content of superoxide anions, such as SOD, GSH Px, CAT, LDH in the striatum of Parkinson’s mice, while reducing the content of H_2_O_2_, MDA, etc., indicating that SP also has strong antioxidant activity *in vivo*. Histologically, a significant increase in the number of neurons was observed in hippocampal slices of the mouse brain tissue, with larger cell bodies, denser and more organized arrangement, reduced intercellular gaps, and significant restoration of adhesion ability, indicating good recovery of cell morphology and function. Although SP was confirmed to reduce oxidative stress markers, like SOD, GSH-Px, CAT, MDA, etc. in both *in vitro* and *in vivo* experiments, the study failed to detect core pathological markers of PD, such as the loss of dopaminergic neurons in the substantia nigra, thereby rendering the neuroprotective conclusions indirect. In summary, polysaccharides can enhance the antioxidant enzyme activity in the striatum of mice and protect dopamine neurons in the brain.

In a study on crude polysaccharides from Chroogomphis rutilus (Zhang et al., 2011) showed that they can inhibit lipid peroxidation in the liver of MPTP injected PD model mice. The results showed that the minimum antioxidant activity of crude polysaccharides from Chroogomphis rutilus was between 2 and 4 mg/mL. With increasing concentration, the antioxidant activity of polysaccharides from Chroogomphis rutilus was significantly enhanced, even reaching 70% of the antioxidant activity of vitamin C. The number of TH positive cells in the substantia nigra of mice in the Chroogomphis rutilus group increased with the increase of polysaccharide concentration, significantly higher than that in the PD model group. Behavioral observations showed significant improvement in the climbing, swimming, and hanging tests compared to the PD group. These results indicate that polysaccharides from Chroogomphis rutilus have good antioxidant activity, ameliorate injury to dopaminergic neurons, and improve the behavioral status of experimental animals. However, the article did not further explore the specific antioxidant mechanism of polysaccharides from Chroogomphis rutilus. This study conducted detailed behavioral observations and comparisons in mice, but the small sample size may compromise the statistical power. Additionally, the assessment of antioxidant capacity relied solely on hepatic lipid peroxidation assays, without evaluating critical antioxidant enzyme activities or intracerebral ROS levels.

### 5.3 Improve mitochondrial functions

Mitochondria play a central role in cellular energy metabolism (Morris, et al., 2024), providing energy to cells through oxidative phosphorylation. Their regulation of cellular bioenergetic states critically determines whether cells survive or undergo apoptosis. Notably, mitochondrial dysfunction constitutes one of the early pathogenic drivers in PD progression. Experimental models demonstrate that MPTP undergoes bioactivation to MPP^+^, which is selectively internalized via dopamine transporters into dopaminergic neurons. This metabolite potently inhibits mitochondrial complex I activity, precipitating bioenergetic failure and ultimately provoking dopaminergic neurodegeneration with PD-like symptoms. Under physiological conditions, mitochondrial-derived ROS maintain cellular homeostasis. However, pathological ROS accumulation during cellular stress induces oxidative damage to biomolecules, such as proteins, DNA, and RNA, thereby exacerbating mitochondrial impairment. Crucially, mitochondrial morphology and functionality exhibit tight coupling. Both exogenous stressors and intracellular oxidative stress disrupt mitochondrial morphology and bioenergetic function. The reticular network within mitochondria represents the integrity and damage of organelle health manifests as: (1) abnormal grid morphology, (2) elevated membrane potential (Δψm), (3) aberrant opening of mitochondrial permeability transition pores (mPTP), (4) altered inner membrane permeability, and (5) depressed enzymatic activity, collectively culminating in functional deficits. The mitochondrial membrane potential is also affected by the stimulation of cell apoptosis. After receiving the apoptosis signal, a decrease in mitochondrial membrane potential can be observed in the early stage, and membrane permeability increases, thereby establishing a pro-apoptotic cascade. The excessive accumulation of ROS leads to structural damage marked by mitochondrial membrane potential collapse, which in turn triggers the release of Cyt C from mitochondria, ultimately inducing apoptosis.

Mitochondrial function is finely regulated by various upstream regulatory factors (He et al., 2023), including: AMPK, p38 MAPK, SIRT1. Peroxisome proliferators activated receptors gamma coactivator one alpha (PGC-1α) is key gene regulating mitochondrial biogenesis. It can activate various transcription factors, such as nuclear respiratory factor-1/2 (NRF-1/2), estrogen-related receptors (ERRs), and PPARγ, promoting mitochondrial function and energy metabolism.

Polysaccharides from TCM protect mitochondrial morphology and structure. A study on tricholoma matsutake polysaccharides (TMP) (Lv et al., 2024) demonstrated that the TMP-H (high concentration of TMP) group and TMP-M (medium concentration of TMP) group showed an increase in the average length and number of mitochondrial network branches, as well as an increase in mitochondrial membrane potential (ΔΨm), which stabilized the morphology of the mitochondrial network and significantly increased the cell survival rate and the number of cell body protrusions. This indicates that TMP can repair MPP + - induced injury to the mitochondrial network morphology in PC12 cells, stabilize mitochondrial membrane potential, and protect mitochondrial morphology and structure.

6-OHDA causes an increase in ROS, which leads to damage and a decrease in mitochondrial membrane potential (Δ ψ m), while sea cucumber polysaccharides (Cui et al., 2016) can effectively reduce ROS levels, stabilize mitochondrial membrane potential, and protect mitochondrial structure from damage. It can also activate the PI3K/Akt signaling pathway, which plays a crucial role in maintaining mitochondrial function and cell survival. Activation of this pathway helps to inhibit cell apoptosis and protect normal mitochondrial function. In addition, sea cucumber polysaccharides (Cui et al., 2016) can inhibit the activation of NF- κB, thereby reducing the expression of iNOS and the production of NO, which helps protect mitochondria from oxidative stress injury.

Fucoidan protected mitochondrial function through the PGC-1 α/NRF2 signaling pathway, which enhances antioxidant enzyme activity to improve mitochondrial antioxidant capacity and maintain normal mitochondrial function. Mitochondrial respiratory capacity is a critical determinant of cell survival. Rats treated solely with rotenone exhibited significantly reduced mitochondrial respiratory function, including basal respiration, ATP production, and maximal respiration. Fucoidan at 140 mg/kg completely restored the levels of all three parameters (Zhang et al., 2018), indicating a protective effect on the mitochondrial respiratory chain. In a study on LBP (Dai et al., 2022), it was reported that LBP demonstrated a protective effect on mitochondrial respiratory function. It alleviated the increase of mitochondrial oxidative respiratory enzyme I in the striatum cells of PD rats induced by rotenone, causing only reversible and mild mitochondrial edema, and alleviating mitochondrial damage in the substantia nigra and striatum of PD rats.

Research on the mechanisms of polysaccharides from the sea cucumber is relatively comprehensive. However, the high cost of obtaining it may limit their clinical application. While these studies emphasize mitochondrial protection, deeper mechanistic links (e.g., direct binding targets of polysaccharides) and *in vivo* pharmacokinetics remain underexplored. Future studies could further investigate whether polysaccharides exert their effects by inhibiting mitophagy (via the PINK1/Parkin pathway, etc.) or modulating endoplasmic reticulum stress (through the PERK/eIF2α pathway, etc.). Comparative analyses of polysaccharide efficacy across models could strengthen translational relevance.

### 5.4 Induction of autophagy and inhibition of aggregation of abnormal proteins

Autophagy refers to the process in which cells engulf, degrade, and recycle damaged and aging organelles, clearing abnormal protein aggregates to maintain cellular homeostasis. Autophagy is crucial for maintaining neuronal metabolism. Dysfunction of autophagy can lead to the accumulation of pathological proteins that can not be cleared and cause neurotoxicity, which is considered one of the important mechanisms for inducing PD. The AMPK/mTOR signaling pathway is currently widely recognized as an autophagy regulatory signaling pathway. AMPK is an important energy receptor in cells. Under conditions, such as hunger and hypoxia, the AMP/ATP ratio increases, promoting AMPK phosphorylation and activation (Yang L. C. et al., 2018). Conversely, mTOR is a member of the PI3K protein kinase family and a negative regulator of autophagy. It can inhibit the activity of ULK1-Atg13-FIP200 initiation complex, a key target of cellular autophagy. Activated AMPK converts mTOR to an inactive p-mTOR state. Consequently, its inhibitory effect on autophagy is removed and autophagy is induced (Liang et al., 2024). The PI3K/AKT/mTOR signaling pathway is involved in the regulation of autophagy, with PTEN serving as a negative regulator of PI3K. After PI3K is activated, it promotes the conversion of PIP2 to PIP3 and phosphorylates Akt. The activated Akt directly activates the mammalian target of rapamycin complex 1 (mTORC1), which can inhibit the initiation stage of autophagy. The activated mTOR can inhibit autophagy by affecting the formation of the mTORC1 complex. LC3І/П and Beclin are autophagy markers and are often used to evaluate autophagy levels.

The experimental results of rotenone-induced PD rat model showed that the p-AMPK/AMPK ratio in the striatum of rats in the PD group was significantly decreased, and LBP significantly reversed this decrease in the p-AMPK/AMPK ratio in the striatum, increased the phosphorylation level of AMPK and the autophagy level (Dai et al., 2022). These indicate that LBP can regulate autophagy through the AMPK/mTOR signaling pathway, eliminate abnormal proteins, alleviate injury to neurons caused by the accumulation of abnormal proteins, and slow down the PD progression.

To determine whether LBP regulates autophagy through the AKT/mTOR pathway, Western blot analysis was conducted to assess the protein levels of p-AKT and p-mTOR in different groups. It was found that levels of phosphorylated AKT and mTOR in the substantia nigra of mice decreased after MPTP treatment. However, LBP attenuated this decrease of p-Akt and p-mTOR (Wang et al., 2018). Further studies found that the protein expression of PTEN also decreased after LBP treatment. This indicates that LBP treatment exerts its neuroprotective effect and alleviates neuronal damage caused by abnormally aggregated α-syn by inhibiting PTEN and regulating excessive autophagy of dopamine neurons through the AKT/mTOR pathway.

**FIGURE 3:**
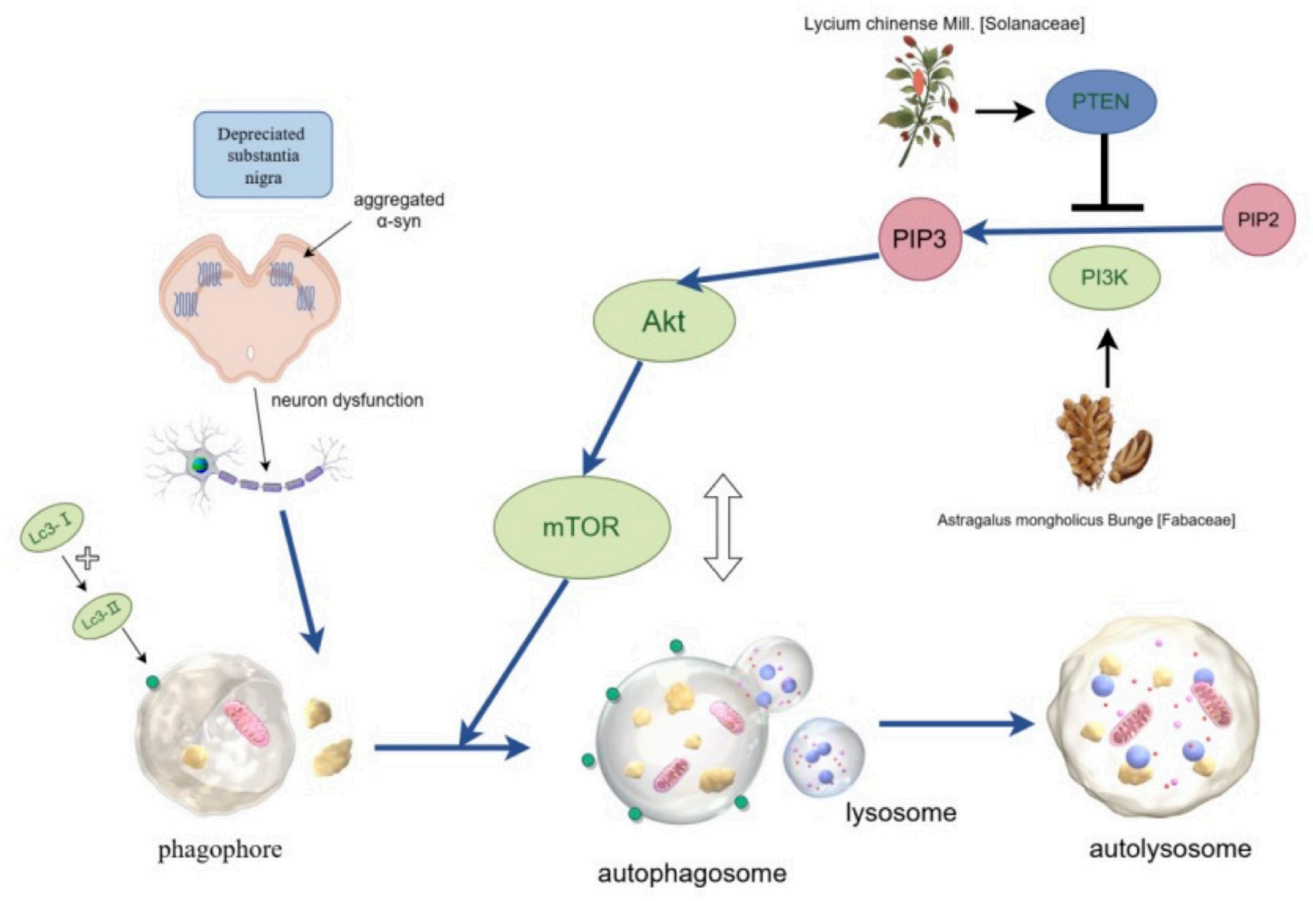
LBP and APS regulate autophagy through the PI3K/AKT/mTOR pathway. The activity of signaling pathway is regulated by phosphorylation of AKT and mTOR, and PTEN is a negative regulator. This pathway has a bidirectional regulatory effect on autophagy: it can maintain the basic level of autophagy while avoiding excessive autophagy.

In a study on APS, it was reported that APS could promote the transformation from LC3-I to LC3-II, simultaneously downregulate the expressions of pAKT and pmTOR, upregulate the expression of PTEN, and increase cell viability and autophagy. When the PI3K protein in PC12 cells is knocked down (KD), the effect of APS is reversed in PI3K KD cells (Tan et al., 2020). It indicates that APS activates autophagy through the PI3K/AKT/mTOR pathway, thereby combating PD. Super-resolution microscopy (SIM) and transmission electron microscopy (TEM) observed that the formation of autophagosomes increased in PC12 cells treated with APS, while the formation of autophagosomes decreased in PI3K KD cells. This further confirms that APS regulates autophagy levels through the PI3K/AKT/mTOR pathway, improves cell proliferation and exerts anti-PD effects. These two conclusions seem to be contradictory. LBP suppresses autophagy, while APS activates it, yet both have been reported to possess anti-PD effects. This paradoxical phenomenon likely arises from the dual-phase role of autophagy in PD pathogenesis: moderate autophagy clears α-synuclein aggregates, whereas excessive autophagy triggers neuronal apoptosis. The regulatory direction (suppression vs activation) is contingent upon the cellular damage stage and microenvironmental context. However, the underlying mechanisms require further investigation to elucidate the precise molecular basis. LBP and APS regulate autophagy through the PI3K/AKT/mTOR pathway (Figure 3).

Oligomers formed by abnormal aggregation of α-syn manifest as the primary cytotoxic species targeting dopaminergic neurons (Sun et al., 2023). These insoluble aggregates preferentially accumulate within midbrain substantia nigra neurons, forming characteristic Lewy bodies - a cardinal histopathological hallmark of PD. Such pathological inclusions directly trigger caspase-mediated apoptotic cascades and neuronal demise. A study showed that the level of aggregated α-syn in the substantia nigra of mice injected with MPTP was significantly increased. LBP significantly reversed this increase (Wang et al., 2018), confirming that LBP can effectively inhibit the aggregation of α-syn.

The ThT fluorescence kinetics experiment is a classic experiment used for analyzing amyloid protein aggregation. The intensity of fluorescence is proportional to the number of protein β-folded structures. Li (2020) first incubated in the α-syn solution to explore the effect of lentinan (LNT) on the aggregation behavior of α-syn. The results showed that the ThT fluorescence signal of the α-syn group increased over time, indicating that the content of β-folding structures in the α-syn group increased with the extension of time. After adding LNT to the α-syn solution, the fluorescence signals of the samples increased only slightly over time. When the LNT concentration rose to 0.8 mg/mL, the fluorescence intensity was only 5% of that in the α-syn group. This result indicates that LNT can effectively inhibit the generation of β-folded structure substances by α-syn. Further research found that LNT can also trigger collective fragmentation and dispersion of the already aggregated α-syn fibers in the sample, and the protein fiber structure was gradually destroyed and finally disappeared, indicating that LNT can depolymerize α-syn fibers. When cells containing α-syn were incubated with a high concentration of LNT (0.8 mg/mL), the cell survival rate was increased by 30%, indicating that the high concentration of LNT reduced the cytotoxicity of the α-syn complex. In conclusion, LNT can counteract the toxicity of α-syn from three aspects: inhibiting the aggregation of α-syn, deaggregating α-syn, and reducing the toxicity of α-syn. This study pioneers the use of LNT to modulate α-synuclein (α-syn) aggregation and explores novel properties of LNT. However, whether LNT eliminates α-syn aggregates through autophagy induction requires further experimental validation.

### 5.5 Alleviation of neuroinflammation

Neuroinflammation plays an important role in the pathogenesis of PD. It mainly involves the activation of glial cells, the release of inflammatory factors, and the involvement of the peripheral immune system. Among them, inflammation of the central nervous system is mainly due to activation of microglia, as well as astrocytes. The peripheral immune response includes infiltration of T cells, dysfunction of regulatory T cells, and abnormal activation of neutrophils. Microglia exhibit two phenotypes after activation: pro-inflammatory M1 type and anti-inflammatory M2 type. It is worth noting that in PD patients, there is a significant increase in M1 type microglia, and activated M1 type cells can release various pro-inflammatory factors, including chemokines (Joers et al., 2016). These inflammatory mediators (such as TNF α, IL-1 β, IL-6, IL-2, IL-4) and enzymes (such as iNOS and COX-2) orchestrate a self-perpetuating cascade through NF-κB and JAK-STAT signaling pathways, simultaneously compromising blood-brain barrier (BBB) integrity via matrix metalloproteinase-9 (MMP-9) mediated tight junction degradation. Activated microglia release chemokines, facilitating more immune cells to participate in the inflammatory response. Peripheral immune cells (particularly T cells and B cells) migrate to the central nervous system. This process establishes bidirectional crosstalk between brain and peripheral inflammation, thereby synergistically promoting the progression of PD. These pro-inflammatory cytokines induce neuronal damage through the following pathways (Shao et al., 2020): pro-inflammatory cytokines (such as TNF - α and IL-1 β) can directly destroy the structure and function of neurons. Induced oxidative stress, excessive production of ROS and reactive nitrogen species (RNS) leads to oxidative damage to neurons. Inflammatory factors activate the MAPK and NF-κB signaling pathways, promote the misfolding and aggregation of α-syn, and cause a decrease in the number of dopaminergic neurons. Combined effects reduce TH + neurons in substantia nigra pars compacta, accelerating Lewy pathology progression.

In an APS study on PD rats, the therapeutic effect and mechanism of APS on PD in a rat model induced by 6-OHDA was investigated. The study adopted a random grouping design, dividing the experimental rats into the following four groups: PD model group, PD model + L-DOPA group (positive control group), PD model + APS group and a normal control group. It was found that the number of rotations in the PD model + APS group and the PD model + L-DOPA group decreased by 20%, 11%, and 16%, respectively, on the first, seventh, and 14th day. Furthermore, the rotational speed shows non-uniformity, and the maximum rotational speed was reduced to 5–7 r/min (Chen and Zhou, 2015). This result indicates that APS exhibits good therapeutic effects in terms of behavior and are comparable to the efficacy of L-DOPA. Further exploration of the mechanism revealed that the expression levels of inflammatory cytokines IL-6, TNF and IL-2 in the brain tissue of rats in the PD model group + APS group were significantly lower than those in the PD model group on the 14th day. These suggest that APS may exert therapeutic effects on PD by reducing the levels of pro-inflammatory cytokines in the brain and improving the neuroimmune inflammatory response of the body.

It was reported by another study that Polygonatum sibiricum polysaccharide (PSP), when intragastrically administered for six consecutive weeks to 6-OHDA induced PD model rats, increased the expression of PPAR-γ in the brain tissue of PD model rats at the eighth week (Chen et al., 2010). PPAR-γ, a ligand-dependent nuclear hormone receptor, belongs to the nuclear hormone receptor superfamily and plays an important role in regulating gene expression. PPAR-γ has the potential to delay the course of neurodegenerative diseases, such as PD and Alzheimer’s disease, by regulating the production of inflammatory factors. This study indicates that the therapeutic effect of PSP may be related to up-regulating PPAR-γ expression and inhibiting the generation of inflammatory factors.

Comparative analysis reveals both APS and PSP target neuroinflammation through distinct mechanisms: (1) APS directly suppresses cytokine production (IL-6/TNF-α/IL-2), whereas (2) PSP activates PPAR-γ-mediated transcriptional regulation. These findings position plant-derived polysaccharides as multi-target neuroprotective agents against PD-associated neuroinflammation.

The NLRP3 inflammasome plays an important role in neuroinflammation (Paik et al., 2021). NLRP3 is activated by sensing pathogen-associated molecular patterns (PAMPs) and damage-associated molecular patterns (DAMPs). After activation, it recruits ASC and forms the NLRP3 inflammasome with caspase-1. The activated caspase-1 cleaves the precursors of IL-1β and IL-18, releasing mature pro-inflammatory factors, and induces pyroptosis. In PD model mice, Dendrobium huoshanense polysaccharide (DHP) (Shang et al., 2023) significantly improved the motor ability, including the performance in the balance beam experiment, suspension experiment and rod rotation experiment. The mechanisms include: alleviating the injury of dopaminergic neurons in the substantia nigra of the midbrain and increasing the expression of tyrosine hydroxylase (TH); inhibiting the inflammatory response by reducing the levels of pro-inflammatory factors IL-1β, TNF-α and IL-18 in microglia; reducing the expressions of NLRP3, ASC, Caspase-1 and IL-1β, thereby inhibiting the activation of the NLRP3 inflammasome and reducing the release of pro-inflammatory factors. Antrodia polysaccharides (Yang Y. et al., 2018) also had a similar effect through reducing the expression of pro-inflammatory factors IL-1β, TNF-α and IL-6, inhibiting the excessive activation of microglia, down-regulating the expression of NLRP3-Caspase1 inflammasome and its downstream inflammatory factors, and alleviating the injury to dopaminergic neurons.

These polysaccharides achieve their therapeutic benefits primarily by inhibiting the activation of the NLRP3 inflammasome pathway. Both studies employed ELISA to quantify inflammatory cytokines (IL-1β↑, IL-4↓, IL-6↑) in murine brain tissues, coupled with Western blot analysis of pro-caspase-1, activated caspase-1, and NLRP3 expression. These approaches provided reliable quantification of CNS neuroinflammation and inflammasome activation status. However, the mechanistic interpretations remain correlative, failing to elucidate whether polysaccharides directly bind to NLRP3 or modulate upstream signaling pathways (e.g., NF-κB priming or K^+^ efflux).

Polysaccharides in TCM regulate signaling pathways and mitigate neuroinflammation. TNF-α, IL-1, and the NF-κB pathway can trigger the activation of the NLRP3 inflammasome, and the latter promotes the synthesis and secretion of pro-inflammatory factors. A high-dose of Pholidota chinensis lindl polysaccharides significantly reduced the expression levels of TNF-α, IL-1β and NF-κB in rats, and increased the number of TH-positive neurons (Zhou N. et al., 2021). These indicate that Pholidota chinensis lindl polysaccharides inhibit the activation of the NLRP3 inflammasome by regulating the NF-κB pathway and exerts anti-inflammatory and neuroprotective effects. Momordica charantia polysaccharides (MCPs) can reverse the increase of TLR4, MyD88, and p-p65 proteins induced by MPP+. MCPs inhibit the generation of NLRP3 inflammasome through the TLR4/MyD88/NF-κB signaling pathway and exert an anti-neuroinflammatory effect (Guo et al., 2021).

### 5.6 DA neuroprotection

In recent years, significant progress has been made in research on dopaminergic neuroprotection, particularly in revealing the relationship between cellular aging and neurodegenerative diseases. Research has shown that aging and death of dopaminergic neurons in the substantia nigra are the core pathological mechanisms of PD. Senescence markers, such as β-galactosidase (β-gal), telomerase, p16INK4a, matrix metalloproteinase-3 (MMP-3), and nuclear membrane protein Lamin B1, are closely related to the cellular aging process in PD (Zhang Y. et al., 2023). As a classic marker of cellular aging, β-gal elevation mirrors lysosomal stress in senescent neurons. Telomerase preserves telomeric TTAGGG repeats, delaying replicative senescence. p16INK4a enforces G1 cell cycle arrest via CDK4/6 inhibition, while MMP-3 degrades extracellular matrix components, disrupting neuron-glia crosstalk. Lamin B1 plays a crucial role in maintaining nuclear structure and cell cycle regulation, and its reduction may be associated with neuronal aging and degeneration.

In terms of signaling pathways, the Wnt1 and β-catenin pathways in the substantia nigra are involved in processes, such as cell proliferation, differentiation, migration, and maintaining tissue homeostasis. In MPTP induced PD animal models, activation of the Wnt/β-catenin signaling pathway can improve nigrostriatal injury and reduce the death of dopaminergic neurons. *In vitro* experiments have also shown that activated Wnt signaling pathway can improve MPTP induced dopaminergic neuron cytotoxicity (Wang et al., 2007). GSK-3 β is a key regulatory factor in the Wnt/β - catenin signaling pathway, and its phosphorylation level directly affects the stability and pathway activity of β - catenin.

DHP may protect neurons by delaying the aging of astrocytes. Compared with the normal control group, mice treated with DHP exhibited the following characteristics: the proportion of cells in the G0/G1 phase decreased, the proportion of cells in the S phase increased, the positive rate of β-gal decreased, the expression of telomerase increased, the expressions of p16INK4a and MMP-3 decreased, and the fluorescence expression of Lamin B1 in mice of the DHP 100 mg/kg group and the DHP 350 mg/kg group increased (Zhang P. et al., 2023). All these were related to cellular senescence. These results indicate that DHP can inhibit astrocyte senescence through different mechanisms and pathways, thereby alleviating neuronal damage.

In another study, 6-OHDA induced PD rats were treated with Cistanches Herba polysaccharides (CDPS) (Yin et al., 2020). It was found that after CDPS intervention, the autonomous activity of SD rats was improved, shortened swimming static time and the decreased number of autonomous standing activities were restored, and the protein expression of Wnt1 and β-catenin in the substantia nigra was increased compared with the PD model group. The expression of GSK-3β mRNA was inhibited. It is speculated that CDPS may protect dopaminergic neurons by inhibiting the activity of GSK-3β, up-regulating the expression of β-catenin protein, activating the Wnt/β-catenin signaling pathway, promoting the proliferation and differentiation of dopaminergic cells. However, the mechanistic study did not conclusively prove the direct association between β-catenin and neuroprotection. It is necessary to verify the key nodes of the signaling pathway (such as detecting the nuclear translocation of β-catenin) to prove that the neuroprotective effect stems from the activation of the pathway. Moreover, the experimental design lacked reverse verification using a Wnt pathway inhibitor; for instance, employing a GSK-3β activator to counteract CDPS effects would strengthen the mechanistic argument.

Basic fibroblast growth factor (bFGF), an important neuroprotective factor, has been proven to inhibit the degeneration of dopaminergic neurons and promote their survival and functional recovery by activating the PI3K/Akt and ERK1/2 signaling pathways. A study found that on the 14th day, the TH content in the substantia nigra of rats in the PD model + APS group was significantly higher than that in the PD group (Chen and Zhou, 2015). In addition, the expression level of bFGF protein in substantia nigra was significantly lower than that in the PD model group. The authors interpreted the APS-mediated reduction of the 6-OHDA-induced bFGF increase as suggestive of a direct protective effect on dopaminergic neurons. Nevertheless, the observed decrease in bFGF expression presents a conceptual challenge. As bFGF is a well-established neuroprotective factor, its downregulation would typically be associated with exacerbated damage rather than protection. Therefore, the authors’ speculation that the decrease in bFGF is linked to the neuroprotective effect of APS requires rigorous experimental verification to establish a plausible causal relationship.

In a cell culture study, SH-SY 5Y cells pretreated with crude polysaccharides from Chroogomphus rutilus, followed by a thorough wash and adding MPP + to verify whether they have direct protective effect on neural cells. The results showed that the survival rate of SH-SY 5Y cells pretreated with 800 μg/mL of crude polysaccharides from Chroogomphus rutilus was significantly higher than that of the control group (Zhang et al., 2013). This indicates that the protective effect of crude polysaccharides from Chroogomphus rutilus on MPP + damaged cells stems from the direct protective effect of polysaccharides on cells, rather than the direct combination of polysaccharides with MPP+. This study features a well-designed approach to eliminate interference. By thoroughly washing the cells after polysaccharide pretreatment and before adding the neurotoxin MPP^+^, the study effectively rules out false positives arising from direct binding between the polysaccharides and MPP^+^. However, the research utilizes crude polysaccharides. Without identifying the specific active monomers responsible for the effect (such as determining molecular weight or monosaccharide composition), standardization of the active component remains challenging. Future directions should include: isolating and purifying individual polysaccharide fractions, elucidating their structures using techniques like mass spectrometry (MS) and nuclear magnetic resonance (NMR), and screening these purified components to identify the core active ingredient.

LBP has a significant protective effect on PC12 cells induced by MPP+. A study showed that after 12 h and 24 h of LBP pretreatment, the cell survival rate increased significantly. The increase in cell survival rate was the most prominent after 24 h of pretreatment with 100 μmol/L LBP (Li P. et al., 2020). This indicates that LBP can counteract the neurotoxicity of MPP+ in a concentration-dependent manner and protect dopaminergic neurons.

In a PD silkworm model (Song et al., 2022), it was found that LBP coated on mulberry leaves significantly improved the motor symptoms of silkworms. UPLC detection showed that the dopamine content in the brains of PD silkworms significantly increased after LBP administration. ELISA results showed that LBP significantly enhanced the TH protein level of PD silkworms. Immunofluorescence quantification revealed an increase in the TH protein level, and the mRNA expression levels of downstream DDC and PRKN genes, indicating the protective effect of LBP on the gene expression and synthesis process of dopamine in dopaminergic neurons. This study demonstrates systematic model construction through comprehensive detection methodologies, including motor function assessment via behavioral trajectory analysis, dopamine metabolism quantification using UPLC, oxidative stress evaluation (T-AOC/MDA biomarkers), critical gene expression profiling (TH/PRKN/DDC). However, notable methodological limitations exist: insufficient LBP dosing, with only two doses (50/100 mg/kg) tested, which fails to establish dose-response relationships and an unvalidated positive control, as the LD-CD (levodopa-carbidopa, 4 mM) group used a single concentration without a justification for the dose.

### 5.7 Intestinal flora imbalance and microbial-gut-brain axis regulation mechanism

Recent studies have shown that gut microbial dysbiosis may play an important role in the pathogenesis of PD. Metagenomic profiling reveals PD patients exhibit significant ecological shifts in gut microbiota composition, with a decrease in beneficial bacteria and an increase in harmful bacteria, as well as a decrease in the abundance of gut microbiota characterized by depletion of butyrate-producing taxa (e.g., Faecalibacterium prausnitzii) and concomitant enrichment of pro-inflammatory species (e.g., Enterobacteriaceae), alongside reduced alpha diversity indices. This disruption of gut microbiota may lead to increased permeability of the intestinal epithelial barrier, triggering bacterial and their products (such as lipopolysaccharides) translocation, activating TLR4/MyD88-dependent innate immunity and instigating systemic low-grade inflammation.

In addition, abnormal deposition of α-syn within enteric plexuses precede motor manifestations by 5–10 years, correlating with prodromal gastrointestinal dysfunction. Dysbiosis of gut microbiota can also elevate levels of inflammatory cytokines, such as TNF-α and IL-6, which in turn activate gut glial cells and promote misfolding of α-synuclein. Retrograde propagation studies validate prion-like α-synuclein trafficking along vagal afferents, culminating in nigrostriatal dopaminergic denervation.

Active polysaccharides can be used by intestinal flora as the main energy source, and the degraded monosaccharides promote the colonization of probiotics and maintain the homeostasis of intestinal flora. *In vitro* fermentation studies showed that Dendrobium officinale polysaccharides increased the abundance of some beneficial bacteria in the gut, including *Lactobacillus*, Faecalibacterium and Rummeliibacillus, while reducing the abundance of some harmful bacteria, such as Shewanella, Geodermatophilus, Peptostreptococcaceae, and *Mycobacterium* (Peng et al., 2023). Polygonum polysaccharide can regulate the composition of intestinal microorganisms in mice and enhance the integrity of intestinal barrier (Jiang et al., 2024). This regulatory effect helps to improve the balance of the gut microbiome, thereby promoting gut health.

In the process of polysaccharide degradation, intestinal flora produced a large amount of short-chain fatty acids (SCFA), which reduced the release of intestinal pro-inflammatory cytokines like TNF-α and IL-6, and inhibited intestinal inflammation. Dendrobium officinale polysaccharides increased the content of short-chain fatty acids, such as acetic acid, propionic acid and butyric acid in the gut, which improved intestinal health (Fu et al., 2019).

Mediated by enteric nervous system inflammation, microglia are abnormally activated, promoting the release of IL-1β, TNF-α, IL-6, NO and proteases in the brain, inducing neuroinflammation and eliciting injury to dopaminergic neurons. Dendrobium officinale polysaccharidesdown-regulated the expression of pro-inflammatory cytokines, such as TNF-α, IFN-γ, IL-4 and IL-6, and increased the expression of anti-inflammatory cytokine IL-10, improved gut microbiome mediated immune response and restored gut flora (Liang et al., 2022).

### 5.8 Multifunctional mechanisms of polysaccharides in PD treatment

#### 5.8.1 Regulation of neurotransmitter systems

PD patients not only have degeneration of DA neurons, which affects motor regulation (such as gait and balance control), but also demonstrate strong associations with dementia-related cognitive decline. The cholinergic neurons also show reduced expression of N-acetylcholine receptor (nAChR) α4β2 subtypes along with upregulated M-acetylcholine receptor (mAChR) M1/M4 subtype activity (Golan et al., 2022; Ni, 2023).

Acetylcholine (ACh) plays a crucial role in regulating behaviors, such as movement, foraging, mating, and egg laying in *C. elegans*. The experiment on the regulatory effect of APS on acetylcholinesterase (AChE) activity in BZ555 nematodes showed that BZ555 nematodes exposed to 6-OHDA exhibited significant downregulation of AChE activity. However, when exposed to a solution containing 2.0 mg/mL APS, its AChE activity and behavioral activity was significantly normalized (Li et al., 2016). It is speculated that the protective effect of APS may be related to protecting AChE from damage and restoring acetylcholine system function. APS effectively inhibits the neurotoxicity caused by 6-OHDA, thus playing a neuroprotective role at the system level. This study showed concentration-dependent neuroprotection within the range of 0.5–4.0 mg/mL APS with 4.0 mg/mL being the optimal concentration. However, nematodes lack the blood-brain barrier, immune system, and complex neural circuits of mammals, making it impossible to directly translate these findings to human physiology.

#### 5.8.2 Behavioral improvement in PD models

Accumulating preclinical evidence demonstrates that botanical polysaccharides exhibit significant motor improvement effects in a mouse model of PD. Among them, LBP ameliorates PD-like symptoms including hypophagia, gait instability, and static tremors, as well as motor coordination, such as pole climbing and grasping. LBP can significantly improve cognitive deficits in Morris Water Maze Test and significantly shorten their escape latency (Wang et al., 2007). ASP can significantly improve the limb grip and suspension dwell time of PD mice, and alleviate motor dysfunction (Zhang et al., 2024). PSP showed good performance in gait regulation and paw extension tests, while CDPS significantly increased the spontaneous activity and standing frequency of PD mice. The polysaccharides from Chroogomphis rutilus also showed significant improvement effects in suspension experiments, pole climbing, and swimming tests, further demonstrating the potential value of polysaccharides from TCM in PD treatment.

#### 5.8.3 Suppression of astrocyte activation

The activation of astrocytes is a hallmark of neurodegeneration, mainly manifested by an increase in GFAP levels. MPTP injection into mice resulted in a significant increase in GFAP positive cells, enlarged cell spheres, and more cell protrusions in the ventral striatum. The LBP treatment group showed a decrease in GFAP, which alleviated the activation of astrocytes in PD mice and exerted its protective effect on dopaminergic neurons.

#### 5.8.4 Multi-target mechanistic actions

Polysaccharides, composed of monosaccharides, usually have unique biological activities. These components often regulate multiple targets in the body and work synergistically. For example, the effect of Stichopus japonicus polysaccharide (SJP) on the SH-SY5Y PD model of human neuroepithelial tumor cells induced by 6-OHDA was investigated. It was demonstrated that SJP inhibited intracellular ROS generation and reduced oxidative stress damage. It inhibited the expression of cyclin D3, leading to the arrest of the cell cycle in the G1/S phase. In the meantime, it reversed the change in the Bax/Bcl-2 ratio and activated both p38/c-Jun N-terminal kinase (JNK)-MAPK and PI3K/Akt pathways, which play a crucial role in cell apoptosis. Furthermore, the activation of NF-κB, preventing the upregulation of iNOS and NO release in cells, and alleviating mitochondrial dysfunction were observed (Wang J. et al., 2021). SJP effectively protects dopaminergic neurons through the above five mechanisms.

Similarly, LBP can exert strong antioxidant effects, effectively suppress α-syn aggregation, and inhibit cell apoptosis through the PI3K/Akt pathway. As mentioned earlier, the mechanism of LBP in anti-PD involves multiple pathways, including: 1) eliminating ROS, reducing oxidative stress, reducing Cyt C release, and anti-apoptosis; 2) inhibiting the increase of mitochondrial oxidative respiratory enzyme Ⅰ in cells and alleviating mitochondrial oxidative damage; 3) enhancing the activity of antioxidant enzymes to combat oxidative stress; 4) inhibiting the aggregation of α-syn and alleviating the damage to DA neurons caused by its toxic effects; 5) activating the PI3K/Akt pathway to resist apoptosis; 6) regulating autophagy in cells through the AMPK/mTOR signaling pathway.

Ganoderma lucidum polysaccharides not only reduces the generation of ROS, upregulates the activity of SOD, alleviates oxidative stress damage, but also reduces the expression of pro-inflammatory factors IL-1 β, TNF α, and IL-6, inhibits excessive activation of microglia, and exerts anti neuroinflammatory effects. They can also alleviate inflammation by downregulating the expression of NLRP3-Caspase1 inflammasome and its downstream inflammatory factors (Yang Y. et al., 2018).

Tricholoma matsutake polysaccharides can stabilize mitochondrial network morphology and membrane potential, protect mitochondrial function, and reduce cell apoptosis by reversing MPTP induced decrease in the Bax/Bcl-2 ratio.

Momordica charantia polysaccharides (MCPs) can reduce the expression of inflammatory factors like TNF - α and IL-1 β, increase the expression of anti-apoptotic protein Bcl-2, and regulate anti-apoptosis. MCPs can also exert anti-inflammatory and anti-apoptotic effects by regulating the TLR4/MyD88/NF-κB signaling pathway. A summary of mechanisms underlying the therapeutic effect of TCM on PD can be found in Table 3.

**TABLE 3:** Mechanisms underlying the therapeutic effect of TCM on PD.

Mechanism Classification	Polysaccharide Name	Test Object	Mechanism/Signaling pathway	Reference
Inhibition of Apoptosis	Lycium barbarum polysaccharides	SH-SY5Y	Downregulate Cyt C and Bax/Bcl-2, caspase-3 and caspase-9; activate the PI3K/Akt signaling pathway, upregulate p-Akt	Wang and Han, 2021; Li P. et al., 2020
	Lycium barbarum polysaccharides	PC12 cells	Inhibit NF-κB signaling pathway, downregulate nNOS and iNOS, Ca^2+^, downregulateCyt C, Bax/Bcl-2, and p38	Gao et al. (2014)
	Angelica sinensis polysaccharides	Mice	Clear ROS, downregulate Bax/Bcl-2, caspase-3	Zhang et al., 2024
	Astragalus polysaccharides	BZ555 nematodes	Downregulate egl-1	Li et al. (2016)
	Sea cucumber polysaccharides	SH-SY5Y	Downregulate Bax/Bcl-2, cl-caspase-9/caspase-9, cl-caspase-3/caspase-3	Wang J. et al. (2021)
Antioxidative Stress	Lycium barbarum polysaccharides	Mice	Increase in SOD, GSH-Px, and CAT activity, decrease in MDA.	Chen et al. (2018)
	Chroogomphus rutilus polysaccharides	Mice	Increase in total antioxidant capacity	Zhang et al. (2011)
	Astragalus polysaccharides	Astrocyte cells	Increase in GSH, GSH-Px, and SOD activity, decrease in MDA.	Li et al. (2006)
	Sulfated Poria cocos polysaccharides	PC12 cells	Increase in SOD, GSH-Px, and CAT activity, decrease in MDA.	Gao et al. (2015a)
	Sulfated Poria cocos polysaccharides	Mice	Increase in SOD, GSH-Px, LDH, and CAT activity, decrease in MDA and H_2_O_2_	Gao et al. (2015b)
	Antrodia camphorata polysaccharides	Mice	Increase in SOD, GSH-Px, and CAT activity, decrease MDA and H_2_O_2_	Yang et al. (2017)
Induction of Autophagy	Lycium barbarum polysaccharides	Rats	Activate the AMPK/mTOR signaling pathway	Dai et al. (2022)
	Astragalus polysaccharides	PC12 cells	Activate the PI3K/AKT/mTOR signaling pathway, downregulate pAKT and pmTOR, upregulate PTEN, LC3-II/LC3-I	Tan et al. (2020)
Inhibition of α-Synuclein	Lycium barbarum polysaccharides	Mice	Inhibit α-synuclein aggregation	Wang et al. (2018)
	Lentinan	α-Syn solution	Inhibit β-sheet formation, depolymerize α-Syn fibers, reduce α-Syn toxicity	Li (2020)
Protection of Mitochondrial Morphology and Structure	Tricholoma matsutake polysaccharides	PC12 cells	Stabilize mitochondrial membrane potential, stabilize mitochondrial structure, downregulate Bax/Bcl-2	Lv et al. (2024)
	Sea cucumber polysaccharides	SH-SY5Y	Reduce ROS levels, stabilize mitochondrial membrane potential, activate the PI3K/Akt signaling pathway; inhibit the NF-κB signaling pathway, downregulate iNOS.	Cui et al. (2016)
Protection of Mitochondrial Respiration Function	Fucoidan	Rats	Enhance mitochondrial respiratory function, activate the PGC-1α/NRF2 signaling pathway	Zhang et al. (2018)
	Lycium barbarum polysaccharides	Rats	Reduce mitochondrial oxidative respiratory enzyme I, upregulate p-AMPK/AMPK.	Dai et al. (2022)
Alleviation of Neuroimmune Inflammation	Astragalus polysaccharides	Rats	Reduce IL-6, TNF, IL-2	Chen and Zhou (2015)
	Polygonatum polysaccharides	Rats	upregulate PPAR-γ expression	Chen et al. (2010)
	Dendrobium huoshanense polysaccharides	Mice	Reduce IL-1β, TNFα, IL-18, MDA, ROS; inhibit NLRP3 inflammasome; increase SOD.	Shang et al. (2023)
	Antrodia camphorata polysaccharides	Mice	Reduce IL-1β, IL-4, IL-6, inhibit NLRP3-Caspase1, NLRP3 inflammasome	Yang L. C. et al. (2018)
	Pholidota chinensis Lindl polysaccharides	Rats	Reduce TNF-α, IL-18, inhibit the NF-κB signaling pathway	Zhou N. et al. (2021)
	Momordica charantia polysaccharides	Mice	Inhibit the TLR4/MyD88/NF-κB signaling pathway, reduce TNF-α, IL-1β, increase in *Bcl-2/Bax*	Guo et al. (2021)
Dopamine (DA) Neuroprotection	Dendrobium huoshanense polysaccharides	Mice	Downregulate the G0/G1 phase cell ratio, increase in the S phase cell ratio, B-gal, p16INK4a and MMP-3; upregulate telomerase expression, Lamin B1	Zhang et al. (2023)
	Cistanche deserticola polysaccharides	Rats	Activate the Wnt/β-catenin signaling pathway, upregulate β-catenin, Wnt1, inhibit GSK-3β	Yin et al. (2020)
	Astragalus polysaccharides	Rats	Downregulate bFGF expression	Chen and Zhou (2015)
	Chroogomphus rutilus polysaccharides	SH-SY5Y	Direct protective effect on cells	Zhang et al. (2013)
	Lycium barbarum Polysaccharides	PC12 cells	Direct protective effect on cells	Li P. et al., (2020)
Regulation of Gut Microbiota Imbalance	Dendrobium officinale polysaccharides	Mice	Increase in beneficial bacteria abundance, decrease in harmful bacteria abundance, downregulate TNF-α, IFN-γ, IL-4, and IL-6, increase in IL-10	Jiang et al., 2024; Liang et al., 2022
	Dendrobium officinale polysaccharides	*In vitro* fermentation	Increase in beneficial bacteria abundance, decrease in harmful bacteria abundance, increase in intestinal short-chain fatty acids	Fu et al. (2019)
Improvement of Neurotransmission	Astragalus polysaccharides	BZ555 nematodes	Activate acetylcholinesterase activity	Li et al. (2016)

## 6 Application of polysaccharides from traditional Chinese medicine

As previously described, Chinese Medicine Prescriptions containing Astragalus, Lycium barbarum, Cistanche deserticola, etc., have been proven to improve symptoms of PD in clinical trials. The mechanisms include regulating the PI3K/Akt/mTOR pathway to inhibit neuronal apoptosis, suppressing caspase cascade reactions, and inhibiting α-syn accumulation. These mechanisms overlap with the action mechanisms of polysaccharides derived from TCM, suggesting the universality of polysaccharide components in the treatment of PD. The strong preclinical evidence for polysaccharides from TCM indicates that they can alleviate motor and cognitive defects in PD mouse models. These findings collectively suggest that polysaccharides from TCM may exert their effects through synergistic mechanisms. Their ability to simultaneously relieve motor and non-motor symptoms in PD models makes them an important candidate in developing comprehensive treatment strategies for neurodegenerative diseases.

At present, hundreds of polysaccharides have been discovered, but due to their high molecular weight, complex structure, and difficulty in purifying bioactive compounds, only seven polysaccharides have been approved to enter the market. Among them, the most widely used are anticoagulant heparin and chondroitin sulfate for the treatment of osteoarthritis. ASP is used for diabetic nephropathy due to its anti-inflammatory effect (Zhou G. et al., 2021), which can ameliorate inflammatory reaction and reduce urinary protein with a good safety profile. Although there is currently no clinical research on polysaccharides in the treatment of PD, it has been confirmed in in vitro cell experiments and animal experiments that polysaccharides from TCM have multiple biological functions, such as immune regulation, antioxidant, lowering blood glucose, and protecting the nervous system. The safety and efficacy of traditional Chinese medicine polysaccharides have been verified.

## 7 Conclusion and future perspectives

In conclusion, polysaccharides derived from TCM demonstrate potential in treating PD through multi-target mechanisms. Although over a dozen of polysaccharides have been shown to enhance cell survival, improve motor function in animal models, and histologically delay PD pathological progression, there remains a notable absence of clinical trials validating their efficacy. Current research faces limitations, such as the vast majority of studies have a relatively small sample size; cellular investigations predominantly focus on signaling pathways, with insufficient depth of exploration. Most literature only detect the expression of key proteins (e.g., Bax/Bcl-2 and caspase-3 expression), lacking exploration of upstream regulators (miRNAs, epigenetic modifications) and pathway crosstalk (e.g., PI3K/Akt-NF-κB interactions); animal experiments primarily employ single neurotoxin models that only partially replicate human PD pathology, failing to capture complex etiological factors like α-syn aggregation and neuroinflammatory cascades.

In the future, network pharmacology can be combined with metabolomics, transcriptomics, and neuroimaging to decipher precise neuroprotective mechanisms of polysaccharides and their interactions. Large-scale randomized controlled trials with extended observation periods are required. Standardized extraction/purification protocols are needed to address compositional variation. Advanced pharmaceutical engineering strategies, such as chemical modifications (sulfation/selenization) to enhance bioavailability, nano-delivery systems for improved blood-brain barrier penetration, can be improved to promote the translation of herbal polysaccharides from basic research to clinical practice and accumulate more high - quality clinical data.

While polysaccharides from TCM exhibit promising multi-target anti-PD properties, their clinical translation requires optimized formulation strategies and high-quality evidence generation to advance from basic research to therapeutic applications.
